# Primary Hyperventilation in the Emergency Department: A First Overview

**DOI:** 10.1371/journal.pone.0129562

**Published:** 2015-06-25

**Authors:** Carmen Andrea Pfortmueller, Sandra Elisabeth Pauchard-Neuwerth, Alexander Benedikt Leichtle, Georg Martin Fiedler, Aristomenis Konstantinos Exadaktylos, Gregor Lindner

**Affiliations:** 1 Department of Anesthesiology and Intensive Care Medicine, Medical University of Vienna, Vienna, Austria; 2 Department of Emergency Medicine, University Hospital and University of Bern, Bern, Switzerland; 3 Center of Laboratory Medicine, University Institute of Clinical Chemistry, Inselspital-Bern University Hospital, Inselspital, Bern, Switzerland; 4 Department of Respiratory and Critical Care Medicine, Otto Wagner Hospital Vienna and Ludwig Boltzmann Institute for COPD and Respiratory Epidemiology, Vienna, Austria; Azienda Ospedaliero-Universitaria Careggi, ITALY

## Abstract

**Background:**

Primary hyperventilation is defined as a state of alveolar ventilation in excess of metabolic requirements, leading to decreased arterial partial pressure of carbon dioxide. The primary aim of this study was to characterise patients diagnosed with primary hyperventilation in the ED.

**Methods:**

Our retrospective cohort study comprised adult (≥16 years) patients admitted to our ED between 1 January 2006 and 31 December 2012 with the primary diagnosis of primary (=psychogenic) hyperventilation.

**Results:**

A total of 616 patients were eligible for study. Participants were predominantely female (341 [55.4%] female versus 275 [44.6%] male respectively, p <0.01). The mean age was 36.5 years (SD 15.52, range 16-85). Patients in their twenties were the most common age group (181, 29.4%), followed by patients in their thirties (121, 19.6%). Most patients presented at out-of-office hours (331 [53.7%]. The most common symptom was fear (586, 95.1%), followed by paraesthesia (379, 61.5%) and dizziness (306, 49.7%). Almost a third (187, 30.4%) of our patients had previously experienced an episode of hyperventilation and half (311, 50.5%) of patients had a psychiatric co-morbidity.

**Conclusion:**

Hyperventilation is a diagnostic chimera with a wide spectrum of symptoms. Patients predominantly are of young age, female sex and often have psychiatric comorbidities. The severity of symptoms accompanied with primary hyperventilation most often needs further work-up to rule out other diagnosis in a mostly young population. In the future, further prospective multicentre studies are needed to evaluate and establish clear diagnostic criteria for primary hyperventilation and possible screening instruments.

## Introduction

Hyperventilation is respiration that exceeds metabolic demands [1,2] and according to Malmberg et al hyperventilaton is defined as a state of alveolar ventilation in excess of metabolic requirements, leading to a decreased arterial partial pressure of carbon dioxide [3,4] sometimes resulting in respiratory alkalosis and an increase in pH [3]. Wheter changes in carbon dioxid always occur comittant with hyperventilation is controversially discussed [3,5].

Hyperventilation may occur as a primarily or secondary result of an underlying medical condition (e.g., metabolic acidosis in renal failure); this study is restricted to primary hyperventilation.

The term “hyperventilation syndrome” was introduced by Kerr et al in 1937 [1,6] and its pathophysiology was described as: stimulation of the autonomic nervous system with consecutive arousal and stimulation of adrenaline secretion [6]. Adrenaline alone is able to stimulate the respiratory and cardiac centres, leading to an increase in heart rate, cardiac output and respiratory rate [6]. The increase in ventilation expels carbon dioxide from the alveolar spaces, resulting in carbon dioxide being shifted from the blood stream [6]. This sudden loss of carbon dioxide leads to a shortage of acid ions and an alkaline shift [6].

Hyperventilation syndrome is a disorder with no widely accepted diagnostic criteria [5,7]. Therefore its diagnosis widely relies on the physician's experience and medical education. Possible diagnostic tools include blood gas analysis or a hyperventilation provocation test [1]. Several studies have indicated that hyperventilation syndrome is widely common [1,6], with a study by Jones et al estimating a prevalence of 9.5% in the general adult population [4]. Despite this, exact data on the prevalence of hyperventilation in EDs is not available.

Although hyperventilation is a widely recognized medical condition, there have been few studies on the population presenting with primary hyperventilation syndrome to the ED. Therefore, the primary aim of this study was to characterise patients diagnosed with primary hyperventilation at our ED.

## Materials and Methods

### Setting

The study was conducted in the ED of a large tertiary care facility in Switzerland, serving about 1.8 million people and treating more than 35,000 cases per year.

### Definition of primary (= psychogenic) hyperventilation

Primary hyperventilation was defined according to Ter Avest et al[5]. It was presumed to be present when an increased respiratory rate (>20 min) was documented at or before the ED visit and when somatic causes explaining the hyperventilation were absent [5].

### Data collection and retrospective survey

Our retrospective cohort study comprised adult (≥16 years) patients admitted to our ED between 1st January 2006 and 31st December 2012 with the primary diagnosis of primary hyperventilation. All patients presenting to the ED with the primary diagnosis of primary hyperventilation during the study period were eligible for study inclusion. Patients were identified using the appropriate search string (hyperventilation, german: Hyperventilation) in the diagnosis field of our computerised patient database (Qualicare Office, Medical Database Software, Qualidoc AG, Bern, Switzerland). Since this medical database allows instantaneous retrieval of past diagnostic reports, discharge summaries, consultations and other relevant medical documents, it was possible to retrospectively analyse the clinical symptoms, diagnostic testing performed and therapeutic procedures initiated in the ED. The following clinical data was extracted from medical records: admission date, admission time, type of admission (walk-in versus emergency medical services), past hyperventilation episodes, symptoms that occurred (dizziness, thoracial pain, paraesthesias, prior stress, fear), count of comorbidities, known psychiatric comorbidity, type of psychiatric comorbidity, referral to emergency psychiatric services, treatment in the ED, hospitalisation, in-hospital mortality and recurrence within 7 days after discharge. Demographic data, such as gender and age, were also assessed. At our Emergency Department (ED), blood gas analysis and exclusion of underlying medical conditions is the standard diagnostic test for hyperventilation syndrome, if any test is performed at all. The hyperventilation provocation test ist not performed. The decision to perform a blood gas analysis was made by the consultant in charge. Office hours were defined by the study team as between 08:00 and 18:00, out-of-office hours as between 18:01 and 07:59 and night time as between 22.00 and 07.00. The diagnosis of primary hyperventilation was confirmed based on clinical information (anamnesis and clinical examination), laboratory and radiological diagnostic results. All medical records were reviewed by two internal specialists and a specialist in emergency medicine. All specialists had to agree independently on the interpretation of the data. Duplicated records, patients with secondary hyperventilation and patients with admissions not related to primary hyperventilation were excluded from the analysis.

### Statistical Analysis

All statistical analyses were performed with SPSS 20.0 Statistical Analysis program (SPSS Inc; Chicago, IL). The data were summarised using descriptive statistics (means, standard deviations, percentages, medians, quartiles and Ns). The differences in patient characteristics were compared using χ^2^tests for categorical variables, and t tests and ANOVA for continuous variables. Multivariable logistic regression was used to identify predictors for diagnosis of hyperventilation without laboratory analysis, with the model including gender, age groups, admission time, type of referral, psychiatric comorbidities and past hyperventilation episodes. All p values were two tailed and at a level of significance of 0.05.

### Ethical Considerations

The study was approved by the Ethics Committee of the Canton of Bern, Switzerland. No individual informed consent was obtained due to the retrospective study design. Patients records were anonymized prior to analysis.

## Results

**Fig 1:**
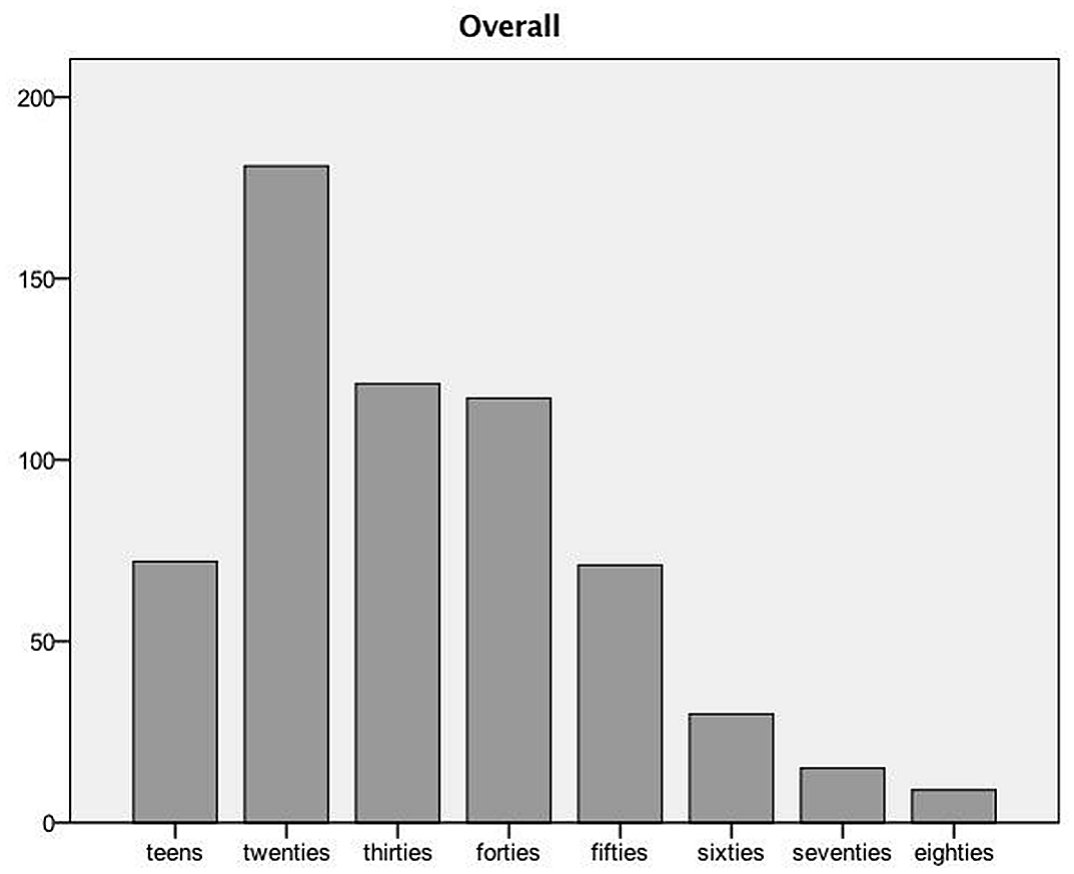
Age distribution.

**Fig 2:**
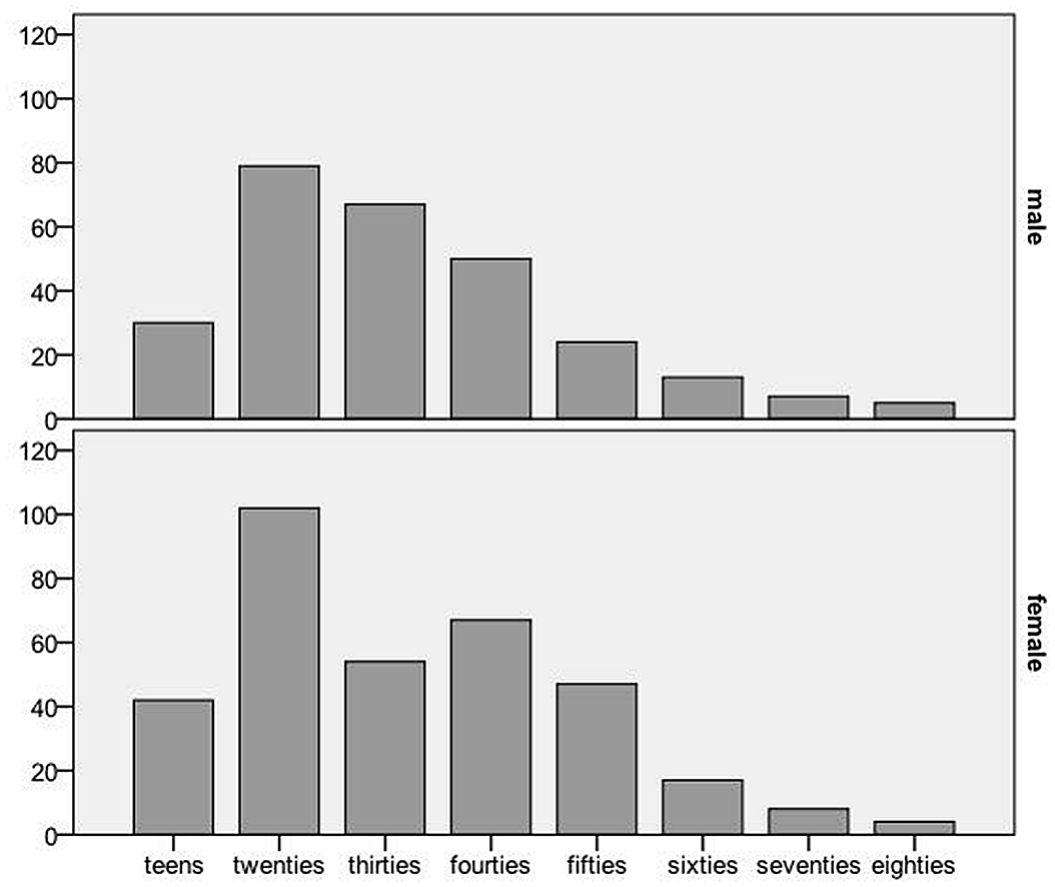
Age distribution.

A total of 616 patients were eligible for study inclusion and an overview of patient characteristics is shown in Table 1. Participants were predominantely female (341 [55.4%] female versus 275 [44.6%] male respectively, p <0.01). The mean age was 36.5 years (SD 15.52, range 16–85). Patients in their twenties were the most common age group (181, 29.4%), followed by patients in their thirties (121, 19.6%). For an overview of age distribution see Figs 1 and 2. Most patients presented at out-of-office hours (331 [53.7%] versus office hours 285 [46.3%], p <0.0001); walk-in patients were most common (422, 68.5%). The most common symptom was fear (586, 95.1%), followed by paraesthesia (379, 61.5%) and dizziness (306, 49.7%). Almost a third (30.4%, 187) of our patients had previously experienced an episode of hyperventilation and half (50.5%, 311) of patients had a psychiatric co-morbidity. The most common psychiatric diagnosis out of all patients with psychiatric co-morbidities was anxiety/panic disorder (122, 39.2%), followed by an acute stress reaction is (133, 36.6%). Almost all patients (593, 96.3%,) were treated as outpatients and no patient died. A total of 244 patients received blood gas analysis at the ED; the results are summarised in Table 2. Mean pH was 7.47, with a maximum of 7.72, while the mean carbon dioxide was 29.76 mmHg with a minimum of 12 mmHg. Three patients (0.5%) were readmitted within 7 days of discharge, all of them suffered from an anxiety disorder.

**Table 1:** Patients Characteristics.

	total (N, %)	arterial blood gas analysis (N, %)	no arterial blood gas analysis (N, %)	p value
**N**	616 (100)	244 (39.6)	372 (60.4)	
Male/female	275 (44.6)/ 341 (55.4)	130 (47.2)/ 114 (33.4)	145 (52.8)/ 227 (66.5)	0.01
age (mean, SD)	36.58 (15.5)	38.18 (16.88)	35.53 (0.75)	0.02
**Nationality**				
Swiss/non-Swiss	410 (66.6)/ 206 (33.4)	168 (41.0)/ 76 (36.9)	242 (59.0)/ 130 (63.1)	0.18
**Admission Time**				
office hours	285 (46.3)	105 (36.8)	180 (63.2)	0.11
night time	183 (29.7)	81 (44.2)	102 (55.7)	0.075
**Referral**				
walk-in/ emergency medical services	422 (68.5)/ 194 (31.5)	157 (37.2)/ 87 (44.8)	265 (62.7)/ 107 (55.2)	0.04
**Symptoms**				
stress	220 (35.7)	91 (41.3)	129 (58.6)	0.28
paraesthesias	379 (61.5)	155 (40.8)	224 (59.2)	0.23
dizziness	306 (49.7)	111 (36.2)	195 (63.7)	0.05
thoracic pain	177 (28.7)	71 (40.1)	106 (59.9)	0.47
fear	586 (95.1)	234 (39.9)	352 (60.1)	0.26
**Past Hyperventilation Episodes**	187 (30.4)	71 (37.9)	116 (62.1)	0.26
**Total Co-Morbidities (SD**, **range)**				
count of co-morbidities	1.89 (0.77, 0–5)	1.89 (0.76)	1.90 (0.78)	0.45
**psychiatric comorbidities (overall)**	311 (50.5)	113 (36.3)	198 (63.7)	0.05
*anxiety/panic disorder*	122 (39.2)	44 (36.1)	78 (63.9)	0.4
*addiction*	18 (5.7)	9 (50.0)	9 (50.0)	0.46
*depression*	34 (10.9)	10 (29.4)	24 (70.6)	0.27
*psychosis*	3 (1.0)	2 (66.6)	1 (33.4)	0.56
*personality disorder*	11 (3.5)	5 (45.4)	6 (54.6)	0.76
*somatisation disorder*	9 (2.8)	5 (55.5)	4 (44.5)	0.32
*acute stress reaction*	113 (36.6)	39 (34.2)	75 (65.8)	0.091
**Referral to emergency psychiatric services**	87 (14.1)	39 (44.8)	48 (55.2)	0.28
**Hospitalisation**				
outpatient/inpatient	593 (96.3)/ 23 (3.7)	229 (38.6)/ 15 (65.2)	364 (61.4)/ 8 (34.8)	0.015
duration of hospitalisation (mean, SD)	6.3 (6.1, 1–25)			
**In-hospital Mortality**	0 (0)	0 (0)	0 (0)	

**Table 2:** Blood gas analysis (n = 244).

	median	First Quartile	Third Quartile
pH	7.47	7.43	7.55
carbon dioxide (pCO_2_)	28.91	23.79	33.98
oxygen (pO_2_)	97.41	96.16	108.83
bicarbonate (Bic)	22.2	20.03	23.97
base excess (BE)	0.00	-0.98	1.50

## Discussion

In the present study on more than 600 patients who received the primary diagnosis of hyperventilation in the ED of a large university hospital, we demonstrate that the syndrome is often associated with fear, paraesthesisas and dizziness.

Even though young people usually are in good health their presentation with symptoms like thoracic pain and dyspnoea make it necessary for the physician to rule out severe medical conditions such as pulmonary embolism or pericarditis. This results in “unnecessary” excessive expenditures leading to a general rise in health care costs. In times were cost stabilisation or reduction is crucial this is problematic. In addtion in our study, most patients (53.7%) presented during out-of-office hours and 29.7% during the night time. Additionally, more than 30% of our patients were referred by emergency medical services. Both factors play an important role for cost management and best resources utilisation. As patients with hyperventilation syndrome may present with diverse, unspecific and sometimes dramatic symptomatology, these patients often meet triage criteria for potentially severe diseases, such as strokes or acute coronary syndrome and are therefore referred to tertiary hospitals with full capacitiy and evaluated as a priority [4]. Given the usually harmless nature of hyperventilation, as confirmed by the very low hospitalisation rates in the present study, the treatment of this population is associated with tremendous costs with little diagnostic output. This problem arises as a result of the lack of established diagnostic criteria for hyperventilation and in the missing evaluated diagnostic screening tools. Therefore it is necessary that further studies should target diagnostic criteria and screening for future pre- or early in-hospital diagnosis of these patients.

To our knowledge this is the first study targeting ED presentations in relation to primary hyperventilation syndrome, making it difficult to place our findings into a broader context. In our population, the incidence of primary hyperventilation syndrome was 0.3% (102 patients/year). This figure probably drastically underestimates the incidence of primary hyperventilation in the general population. According to a Cochrane review by Jones et al on breathing exercises for dysfunctional breathing/hyperventilation syndrome in adults, there is concern that the patients diagnosed with hyperventilation syndrome represent only the tip of the “clinical iceberg”. The aetiology of primary hyperventilation is not yet clear [8], but, as this study suggests, psychological stress may be a triggering factor in the pathogenesis of hyperventilation, as it activates voluntary ventilatory control pathways [8–10]. This would explain why a relatively high percentage of patients had psychiatric comorbidities in this study.

In our study, the most common symptom of hyperventilation syndrome was fear, followed by paraesthesias and dizziness. These symptoms are widely recognized in the medical literature [4,6,8,11]. Fear in particular seems to be a core component of hyperventilation syndrome [2]. Nevertheless, almost thirty percent of our study population presented with thoracic pain/chest tightness. According to Gardner et al, chest tightness is not a symptom of hyperventilation per se [12]. It is therefore important to evaluate these patients for cardiac or other pulmonary causes of thoracic pain, such as pulmonary embolism.

### Limitations

Our study is limited by its retrospective, single centre design. As information in our medical history database is presented in a narrative comment, no guarantee of complete or correct reporting can be given and bias is possible. Additionally, we did not assess precipitating factors for hyperventilations syndrome, such as palpitations, drug abuse etc. and therefore cannot report on these. Furthermore we did not assess whether patients visited our ED again after 7 days which would indicate that the original diagnosis was mistaken. And it is also possible that our patients were admitted to another hospital in the surrounding area or went to a family physician and received another diagnosis there.

Apart from the retrospecitve design the major draw back of the present study is that the diagnosis of In hyperventilation was only based on clinical examination and in some cases on blood gas analysis if any diagnostic evaluation was performed at all. As this was a retrospective describtive study we did not use the hyperventilation provocation test or the Nijmegen questionnaire by van Dixhoorn et al.

Additionally, it would be interesting to know the cost for each ED admission, the diagnostic testing perfomed and the time spent in the ED, although these factors were not analysed. Furthermore, our study was limited to adults (>16 years of age), as children are treated at a separate ED in the same hospital.

## Conclusion

Hyperventilation is a diagnostic chimera with a wide spectrum of symptoms. Patients predominantly are younger fifty, female sex and often have psychiatric comorbidities. The severity of symptoms accompanied with primary hyperventilation most often needs further work-up to rule out other diagnosis in a mostly young population.

In the future, further prospective and multicentre studies will be needed to evaluate and establish clear diagnostic criteria for hyperventilation and possible screening instruments.
